# Coexpression and Secretion of Endoglucanase and Phytase Genes in *Lactobacillus reuteri*

**DOI:** 10.3390/ijms150712842

**Published:** 2014-07-21

**Authors:** Lei Wang, Yuxin Yang, Bei Cai, Pinghua Cao, Mingming Yang, Yulin Chen

**Affiliations:** College of Animal Science and Technology, Northwest A&F University, Yangling 712100, Shaanxi, China; E-Mails: wanglei19831002@gmail.com (L.W.); yxyang@nwsuaf.edu.cn (Y.Y.); caibei1115@gmail.com (B.C.); caopinghua2014@gmail.com (P.C.); ymm@nwsuaf.edu.cn (M.Y.)

**Keywords:** coexpression, secretion, endoglucanase, phytase, *Lactobacillus reuteri*, broiler

## Abstract

A multifunctional transgenic *Lactobacillus* with probiotic characteristics and an ability to degrade β-glucan and phytic acid (phytate) was engineered to improve nutrient utilization, increase production performance and decrease digestive diseases in broiler chickens. The *Bacillus subtilis* WL001 endoglucanase gene (*celW*) and *Aspergillus fumigatus* WL002 phytase gene (*phyW*) mature peptide (*phyWM*) were cloned into an expression vector with the lactate dehydrogenase promoter of *Lactobacillus casei* and the secretion signal peptide of the *Lactococcus lactis* *usp45* gene. This construct was then transformed into *Lactobacillus reuteri* XC1 that had been isolated from the gastrointestinal tract of broilers. Heterologous enzyme production and feed effectiveness of this genetically modified *L. reuteri* strain were investigated and evaluated. Sodium dodecyl sulfate polyacrylamide gel electrophoresis analysis showed that the molecular mass of *phyWM* and *celW* was approximately 48.2 and 55 kDa, respectively, consistent with their predicted molecular weights. Endoglucanase and phytase activities in the extracellular fraction of the transformed *L. reuteri* culture were 0.68 and 0.42 U/mL, respectively. Transformed *L. reuteri* improved the feed conversion ratio of broilers from 21 to 42 days of age and over the whole feeding period. However, there was no effect on body weight gain and feed intake of chicks. Transformed *L. reuteri* supplementation improved levels of ash, calcium and phosphorus in tibiae at day 21 and of phosphorus at day 42. In addition, populations of *Escherichia coli*, *Veillonella* spp. and *Bacteroides vulgatus* were decreased, while populations of *Bifidobacterium* genus and *Lactobacillus* spp. were increased in the cecum at day 21.

## 1. Introduction

In some plant seeds, β-glucan is the main constituent of the cell wall and phytic acid (phytate) is the major storage form of phosphorus [1,2]. They both have a powerful ability to absorb protein and minerals (including P, Ca^2+^, Cu^2+^, Fe^2+^, Mn^2+^, Zn^2+^) to form insoluble complexes or chelate, which may decrease the activity and diffusion of digestive enzymes, hinder absorption of nutrients and stimulate bacterial proliferation [3,4,5]. Phytases could hydrolyze phytic acid to myo-inositol and inorganic phosphate, and endoglucanase could cleave β-linked bonds within the cellulose or glucan molecule to form low molecular weight fragments. However, phytase and endoglucanase are rarely secreted in the digestive tracts of non-ruminant animals. Many nutrients are, therefore, not absorbed and are directly excreted, resulting in low dietary utilization and environmental pollution [6,7,8]. Many studies have demonstrated that supplementation with exogenous enzyme preparations can eliminate anti-nutritional factors and improve nutrient utilization and animal productive performance [9,10,11,12,13,14]. However, these supplements increase feed cost substantially and are only effective in the short term [15,16]. On the other hand, many natural enzymes cannot withstand the high temperatures of the feed pelleting process. A feasible method to overcome these problems might therefore be the development of multifunctional probiotics with degradation activity, achieved by introducing heterologous genes encoding hydrolase [15,17].

*Lactobacilli* have not only received GRAS (generally recognized as safe) status, but have also been used as the delivery vector to express heterologous genes and antigens or as a host with probiotic properties and physiological functions [18]. Furthermore, *Lactobacilli* of animal gastrointestinal tract origin are one of the predominant bacteria in the animal gastrointestinal tract, and have excellent properties of acid and bile salt tolerance as well as a powerful capacity for colonization and adhesion [19]. Thus, it is their unique properties that make expression of heterologous genes in intestinal probiotic strains so promising. Fibrolytic enzyme genes and phytase genes have been cloned and expressed in *Lactobacilli* in several studies [2,16,20,21]. However, coexpression and secretion of phytase and endoglucanase gene products in intestinal *Lactobacilli* has not yet been reported. Therefore, this study aimed to generate a multifunctional transgenic *Lactobacillus* with probiotic characteristics as well as the ability to degrade β-glucan and phytic acid (phytate) for the purpose of improving nutrient availability, increasing production performance and decreasing digestive diseases of broiler chickens. To achieve this we cloned and coexpressed *B. subtilis* WL001 endoglucanase *celW*, and *A.* *fumigatus* WL002 phytase gene (*phyW*) mature peptide *phyWM*, in *L. reuteri* XC1 isolated from the gastrointestinal tract of broiler chickens. The heterologous enzyme production and the broiler feed effectiveness of this genetically modified *L. reuteri* strain were studied.

## 2. Results

### 2.1. Construction of Endoglucanase–Phytase-Coexpressing Plasmids

**Figure 1:**
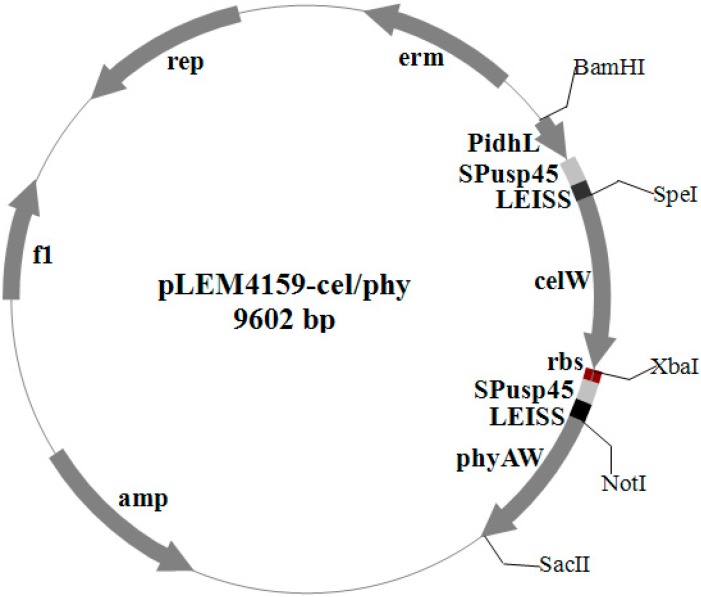
*Lactobacillus* expression plasmid harboring *B. subtilis* WL001 endoglucanase gene *celW*, *A.* *fumigatus* WL002 phytase gene mature peptide *phyWM*, *P_IdhL_* promoter, *usp45* gene signal peptide *SP_usp45_*, enhancer *LEISS* and ribosome binding site *rbs*.

**Figure 2:**
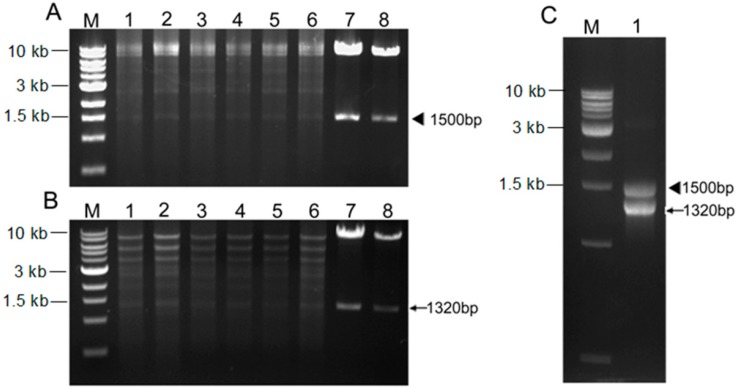
Double-restriction enzyme digestion and PCR analysis of pLEM4159-cel/phy transformants. (**A**) pLEM4159-cel/phy plasmid from *L. reuteri* XC1 or *E. coli DH5α* were double-digested with *Spe*I and *Xba*I; (**B**) pLEM4159-cel/phy plasmid from *L. reuteri* XC1 or *E. coli* DH5α were double-digested with *Not*I and *Sac*II; and (**C**) PCR confirmation of *cel15* and *phyAM* in *L. reuteri* pLEM4159-cel/phy using primer pairs P1/P2 and C1/C2. Lane **M**, 1 kb DNA ladder; Lanes **1**–**6**, *L. reuteri* pLEM4159-cel/phy; and Lanes **7**–**8**, pLEM4159-cel/phy plasmid from *E. coli* DH5α.

The endoglucanase gene *celW* of *B. subtilis* WL001 (1.5 kb *SpeI*–*XbaI* fragment) and the phytase gene mature peptide *phyWM* of *Aspergillus fumigatus* WL002 (1.32 kb *NotI*–*SacII* fragment) were ligated together with a ribosome binding site *rbs* into the *Lactobacillus* expression vector pLEM4156, generating pLEM4159-cel/phy (Figure 1). The recombinant plasmid was transformed into *E. coli* DH5α and verified by double-restriction enzyme digestion (*SpeI*-*XbaI* and *NotI*-*SacII*), which produced a vector fragment (8.0 kb), a *celW* gene fragment (1.5 kb) and a *phyWM* fragment (1.32 kb) (Figure 2A,B, lanes 7 and 8). The polymerase chain reaction (PCR) results of the transformant, *L. reuteri* pLEM4159-cel/phy, indicated that the primer pairs P1/P2 and C1/C2 not only amplified the 1.5 kb *celW* and 1.32 kb *phyWM* fragments, respectively, but also a 2.98 kb fragment representing a tandem arrangement of *phyWM* and *celW* genes (Figure 2C, lane 1). Double-restriction enzyme digestion results of the recombinant plasmid in *L. reuteri* were the same as those in *E. coli* DH5α (Figure 2A,B, lanes 1–6).

### 2.2. Coexpression of Endoglucanase and Phytase Genes in L. reuteri XC1

**Figure 3:**
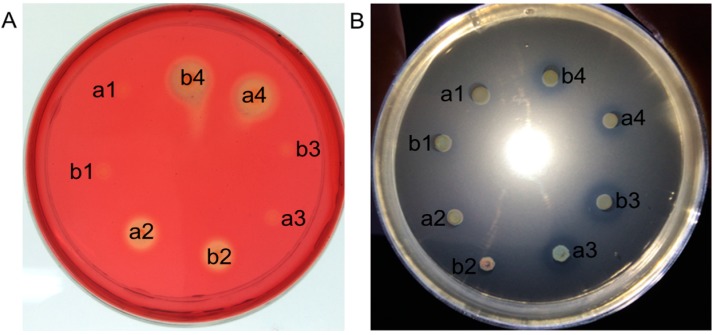
Plate test for endoglucanase and phytase activity of *Lactobacillus reuteri* transformants. Extracellular supernatant (**A**) and intracellular extract (**B**) of growing cells were pipetted into wells of a CMC-containing plate stained with Congo red a and into wells of a sodium phytate-containing plate b. Well **1**, *L. reuteri* pLEM4156; well **2**, *L. reuteri* pLEM4157 (cel); well **3**, *L. reuteri* pLEM4159-cel/phy; well **4**, *L. reuteri* pLEM4158 (phy).

The wild-type *L. reuteri* XC1 and the *L. reuteri* pLEM4156-transformed strains were free of endoglucanase and phytase activity (Figure 3). The culture supernatant and cell lysate of the *L. reuteri* pLEM4157 (cel) strain formed hydrolysis zones on the carboxymethyl cellulose (CMC)-containing plate but not on the calcium phytate containing plate (Figure 3A). In contrast, the culture supernatant and cell lysate of the *L. reuteri* pLEM4158 (phy) strain formed hydrolysis zones on the calcium phytate-containing plate but not on the CMC-containing plate (Figure 3B). The *L. reuteri* pLEM4159-cel/phy strain showed clear halos on the CMC-containing plates and also on the calcium phytate-containing plates, indicating that endoglucanase and phytase were successfully co-expressed and secreted by *L. reuteri* pLEM4159-cel/phy.

As shown in Table 1, the endoglucanase activity in the extracellular fraction of the *L. reuteri* pLEM4157 (cel) culture was 2.5-fold higher than that of the intracellular fraction. Also, approximately 72% of endoglucanase activity was detected in the extracellular fraction. The phytase activity in the extracellular fraction of the pLEM4158 (phy) culture was 1.9-fold higher than that of the intracellular fraction, with more than 66% of total phytase activity present in the extracellular fraction. For *L. reuteri* pLEM4159-cel/phy, not only endoglucanase but also phytase activities were observed in the extracellular and intracellular fractions. The endoglucanase activity of the extracellular and intracellular fractions was 0.68 ± 0.17 and 0.15 ± 0.06 U/mL, respectively. The phytase activity was 0.14 ± 0.16 and 0.42 ± 0.05 U/mL within and outside of cells, respectively. More than 75% of the total endoglucanase and phytase activity of the *L. reuteri* pLEM4159-cel/phy culture was present in the extracellular fraction.

**Table 1:** Activity of endoglucanase and phytase of *L. reuteri* XC1 transformants.

Strain	Enzyme Activity (U/mL) ^1^			
	Endoglucanase		Phytase	
	Intracellular	Extracellular	Intracellular	Extracellular
*L. reuteri* pLEM4156	Nd ^2^	Nd	Nd	Nd
*L. reuteri* pLEM4157 (cel)	0.37 ± 0.09	0.96 ± 0.08	Nd	Nd
*L. reuteri* pLEM4158 (phy)	Nd	Nd	0.26 ± 0.14	0.51 ± 0.13
*L. reuteri* pLEM4159-cel/phy	0.15 ± 0.06	0.68 ± 0. 17	0.14 ± 0.16	0.42 ± 0.05

^1^ One phytase unit was defined as the activity that released 1 μmol of inorganic phosphorous from sodium phytate per minute under the assay conditions. One unit of the fibrolytic enzyme activity was defined as the activity that released 1 μmol of reducing sugar from carboxymethyl cellulose (CMC) per minute under the assay conditions; and ^2^ None detected.

**Figure 4:**
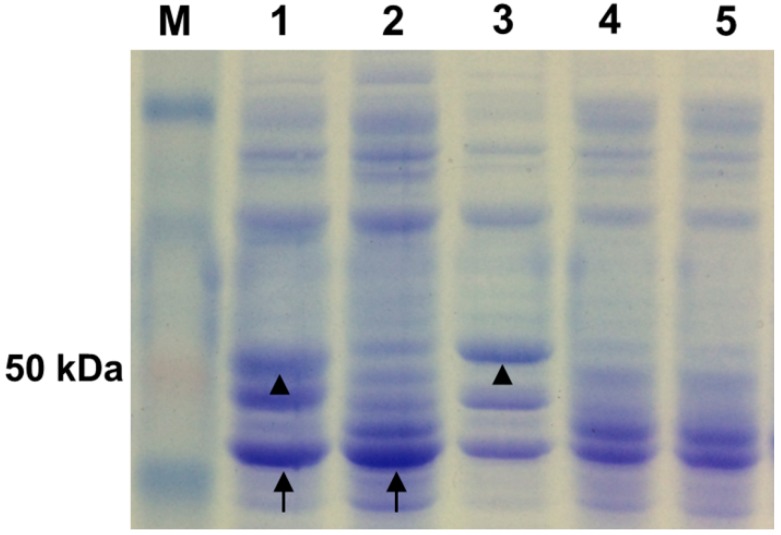
The extracellular supernatant proteins of *Lactobacillus reuteri* transformants were analyzed by SDS-PAGE. Lane **1**, *L. reuteri* XC1; Lane **2**, *L. reuteri* pLEM4156; Lane **3**, *L. reuteri* pLEM4157 (cel); Lane **4**, *L. reuteri* pLEM4158 (phy); Lane **5**, *L. reuteri* pLEM4159-cel/phy; Lane **M**, molecular weight marker. Black triangle: protein band of *celW*; and Black arrow: protein band of *phyWM*.

The endoglucanase and phytase proteins from the supernatant of the transformed *L. reuteri* were analyzed with sodium dodecyl sulfate polyacrylamide gel electrophoresis (SDS-PAGE) to further verify their expression in *L. reuteri*. Coomassie blue staining showed that the molecular mass of *phyWM* and *celW* were approximately 48.2 and 55 kDa, respectively (Figure 4, lanes 3 and 4), which corresponded to the co-expressed molecular weight of *phyWM* and *celW* (Figure 4, lane 5), further confirming that the two genes were efficiently expressed in *L. reuteri*. There were no distinct bands for proteins of about 48.2 and 55 kDa for wild-type *L. reuteri* XC1 or *L. reuteri* pLEM4156 (Figure 4, lanes 1 and 2).

### 2.3. Growth Performance of Broilers

The effect of supplementation of a barley-wheat-based diet with the transformed *L. reuteri* on the growth performance of broilers is presented in Table 2. There were no significant differences in daily body weight, feed intake or feed conversion ratio from 0 to 21 days of age among the treatment groups (*p* > 0.05). There were also no differences in daily body weight and feed intake from 22 to 42 days of age between the treatment groups (*p* > 0.05). However, the feed conversion ratio of chicks from 22 to 42 days of age administered *L. reuteri* pLEM4158 (phy) and *L. reuteri* pLEM4159-cel/phy was significantly lower than that of the negative control group at day 42 (*p* < 0.05). During the second and whole feeding periods, the feed conversion ratio of chicks administered *L. reuteri* pLEM4157 (cel), *L. reuteri* pLEM4158 (phy) and *L. reuteri* pLEM4159-cel/phy was also significantly lower than that of the negative control group (*p* < 0.05).

**Table 2:** Effects of transformed *L. reuteri* strain supplementation on growth performance of broilers.

Performance	Control Group ^1^		*L. reuteri* Group				SEM	*p* Value
	NC	PC	pLEM4156	pLEM4157 (cel)	pLEM4158 (phy)	pLEM4159-cel/phy		
days (1–21)								
BWG, g/bird/day	27.85	28.14	27.83	27.57	28.23	27.32	0.173	0.697
Feed intake, g/bird/day	39.36	39.73	39.26	39.33	39.46	38.75	0.220	0.900
FCR	1.42	1.41	1.41	1.43	1.40	1.42	0.006	0.933
days (22–42)								
BWG, g/bird/day	57.47	62.21	61.46	59.22	61.13	60.66	0.854	0.664
Feed intake, g/bird/day	115.19	115.77	117.47	111.50	112.24	111.07	1.207	0.585
FCR	2.01 ^a^	1.86 ^b^	1.93 ^a,b^	1.89 ^a,b^	1.83 ^b^	1.84 ^b^	0.019	0.049
days (1–42)								
BWG, g/bird/d	42.76	45.31	44.74	43.48	44.77	44.06	0.439	0.598
Feed intake, g/bird/d	77.28	77.75	78.37	75.42	75.85	74.91	0.614	0.532
FCR	1.81 ^a^	1.72 ^b^	1.76 ^a,b^	1.74 ^b^	1.69 ^b^	1.70 ^b^	0.011	0.015

^a,b^ Means within the same row with different superscript letters are significantly different (*p* < 0.05); ^1^ NC, Negative control; PC, Positive control; BWG, Body weight gain and FCR, Feed conversion ratio (feed/gain).

### 2.4. Tibial Parameters of Broiler Chickens

The effect of supplementation of a barley-wheat-based diet with the transformed *L. reuteri* on the levels of ash, calcium and phosphorus in the tibiae of broiler chickens is presented in Table 3. During the second and whole feeding periods, there were no differences in dry weight and length of tibiae (*p* > 0.05). However, at day 21, the ash content of tibiae in chicks administered *L. reuteri* pLEM4159-cel/phy was significantly higher than that in chicks administered *L. reuteri* pLEM4156 or in the negative control group. The ash content in the *L. reuteri* pLEM4159-cel/phy group was not significantly different with those administered *L. reuteri* pLEM4157 (cel), *L. reuteri* pLEM4158 (phy) and the positive control group (*p* < 0.05). The phosphorus content of tibiae in chicks administered *L. reuteri* pLEM4159-cel/phy was not significantly different compared to that in the positive control group (*p* > 0.05). However, compared with the negative control group, the phosphorus levels of tibiae in chicks administered transformed *L. reuteri* had a tendency to increase. The calcium content of tibiae in chicks administered *L. reuteri* pLEM4159-cel/phy and *L. reuteri* pLEM4157 (cel) was significantly higher than that of the negative control group (*p* < 0.05). Compared with the negative control group, all the groups administered *L. reuteri* had slightly increased tibia calcium contents (*p* > 0.05). At day 42, there were no differences in ash or calcium contents of tibiae among the treatment groups (*p* > 0.05); however, the phosphorus levels in tibiae of chicks administered *L. reuteri* pLEM4158 (phy) were higher than those of the negative control group (*p* < 0.05).

**Table 3:** Effects of transformed *L. reuteri* strain supplementation on tibia parameters.

Tibia	Control Group ^1^		*L. reuteri* Group				SEM	*p* Value
	NC	PC	pLEM4156	pLEM4157 (cel)	pLEM4158 (phy)	pLEM4159-cel/phy		
day 21								
Weight, g	0.86	0.71	0.87	0.80	0.78	0.78	0.021	0.317
Length, cm	4.41	4.31	4.52	4.36	4.40	4.30	0.036	0.667
Ash, %	48.47 ^a^	52.31 ^c^	49.15 ^a^	49.46 ^a,b^	50.92 ^a,b,c^	51.71 ^b,c^	0.406	0.016
P, %	9.70 ^a^	10.58 ^b^	9.77	9.75 ^a^	9.91 ^a^	10.13 ^a,b^	0.090	0.030
Ca, %	15.15 ^a^	17.74 ^b^	16.84 ^a,b^	17.82 ^b^	17.14 ^a,b^	18.99 ^b^	0.373	0.044
day 42								
Weight, g	1.99	2.31	2.45	1.98	2.11	2.13	0.060	0.128
Length, cm	6.40	6.74	6.71	6.31	6.36	6.33	0.068	0.128
Ash, %	52.18	50.54	52.36	50.89	52.94	50.84	0.269	0.376
P, %	9.91 ^a^	10.20 ^a,b^	10.10 ^a,b^	10.29 ^a,b^	10.66 ^b^	10.20 ^a,b^	0.081	0.026
Ca, %	17.27	18.09	16.28	20.00	17.68	17.58	0.586	0.473

^a,b,c^ Means within the same row with different superscript letters are significantly different (*p* < 0.05); ^1^ NC, Negative control; PC, Positive control.

### 2.5. Microflora Population in the Cecum

The effect of supplementing the barley-wheat-based diet with the transformed *L. reuteri* on the microflora population in the cecum of broiler chickens is presented in Table 4. At day 21, the *Escherichia coli* population in the cecum of chicks administered *L. reuteri* pLEM4159-cel/phy was significantly lower than that in the positive control group (*p* < 0.05); the *Veillonella* spp. population in chicks administered *L. reuteri* pLEM4157 (cel) was also significantly lower than that in the positive control group. Moreover, the counts of *E. coli*, *Veillonella* spp. and *Bacteroides vulgatus* administered transformed *L. reuteri* indicated a lower trend than those in the control groups. An opposite trend was observed on the counts of *Lactobacillus* spp. and *Bifidobacterium* genus. At day 42, the administration with transformed *L. reuteri* did not affect the microflora populations in the cecum of broilers in comparison to controls, whereas the counts of *Lactobacillus* spp. and *Enterococcus faecalis* in the administered transformed *L. reuteri* groups showed a higher trend than in the control groups.

**Table 4:** Effect of transformed *L. reuteri* strain supplementation on the microflora population in the cecum of broiler chickens (log_10_^c^°^pies^/10 ng DNA).

Groups	Control Group ^1^		*L. reuteri* Group				SEM	*p* Value
	NC	PC	pLEM4156	pLEM4157 (cel)	pLEM4158 (phy)	pLEM4159-cel/phy		
day 21								
*Escherichia coli*	5.44 ^a,b^	6.12 ^b^	4.84 ^a,b^	5.26 ^a,b^	4.70 ^a,b^	2.99 ^a^	0.347	0.05
*Bifidobacterium* spp.	1.50	1.45	1.99	2.19	1.79	2.24	0.116	0.231
*Bacteroides vulgatus*	2.50	3.19	2.31	2.25	2.26	2.07	0.159	0.493
*Veillonella* spp*.*	1.52 ^a,b^	1.82 ^b^	0.96 ^a,b^	0.45 ^a^	0.65 ^a,b^	0.89 ^a,b^	0.170	0.048
*Clostridium* IV	6.34	6.29	5.69	4.94	5.66	6.20	0.237	0.481
*Lactobacillus* spp*.*	3.74	4.44	5.30	4.66	5.18	5.42	0.251	0.322
*Enterococcus faecalis*	1.42	2.20	1.26	2.47	1.23	1.91	0.241	0.594
day 42								
*Escherichia coli*	3.68	3.23	4.51	3.02	3.18	4.04	0.224	0.422
*Bifidobacterium* spp.	0.68	0.72	1.62	1.11	0.94	1.45	0.135	0.295
*Bacteroides vulgatus*	3.39	3.97	2.81	2.51	3.19	4.04	0.331	0.761
*Veillonella* spp*.*	0.50	0.66	0.68	1.57	1.56	1.10	0.174	0.291
*Clostridium* IV	4.47	4.39	5.91	3.91	4.54	5.57	0.300	0.432
*Lactobacillus* spp*.*	3.59	3.04	4.16	4.31	4.31	4.97	0.295	0.531
*Enterococcus faecalis*	0.60	0.39	0.84	1.15	1.25	1.05	0.193	0.802

^a,b^ Means within the same row with different superscript letters are significantly different (*p* < 0.05); ^1^ NC, Negative control; and PC, Positive control.

## 3. Discussion

*Lactobacillus* probiotics have been used to deliver proteins (such as green fluorescent protein (GFP), therapeutic peptides, bacteriocins and enzymes) to the chicken gastrointestinal tract to further the understanding of the mechanisms of colonization and adhesion [22,23], treating disease and cancer [24], substituting antibiotics [25] or improving production performance [26,27]. As a delivery vector, *Lactobacilli* must be able to express and secrete proteins of interest abundantly and efficiently; thus, it is very important to select appropriate and effective transcriptional promoters and secretion signal peptides [28]. Currently, promoters used with *Lactobacillus* are mainly of two types. One type is an inducible promoter, such as the *nisin*-controlled expression system or the *lac*-controlled expression system. The other is a constitutive or native promoter, such as *P_ldhL_* [29], *P_slpA_* [30], *P_144_* [31], *P_23_* or *P_32_* [32]. Compared with constitutive promoters, the expression levels of genes achieved from the inducible promoter controlled systems are higher but the inducible promoter controlled system requires an inducer, which could be degraded by enzymes from the gastrointestinal tract, resulting in increased costs incurred from the addition of the inducer to food or water [28]. A constitutive promoter that can ensure constant production of therapeutic peptides at the site of colonization avoids the limitations described above. Besides, stronger constitutive promoters can be sought to improve expression. *P_ldhL_* has been reported as the most effective promoter for expressing green fluorescent protein in *lactobacillus* [28].

The combination of a homologous signal peptide and a synthetic propeptide has been developed as a new tool to aid secretion [33] of heterologous proteins in *L. lactis* and to facilitate protein purification. The *usp45* gene signal peptide, *SP_usp45_*, with a more consensual structure from *Lactococcus lactis* MG1363, has been fused to a number of mature proteins and peptides to drive their secretion by *L. lactis* [34,35]. *LEISSTCDA* or *LEISS* synthetic propeptide, previously described as a secretion enhancer, efficiently increased the overall heterologous protein production and did not interfere with activity of the heterologous protein [33]. In this study, we expressed endoglucanase and phytase genes in *L. reuteri* using the *P_ldhL_* promoter and the *Sp_usp45_* signal peptide fused with the *LEISS* synthetic propeptide.

*Lactobacilli* are generally considered to be poor secretors [36], but a few heterologous proteins have been expressed and secreted by intestinal *Lactobacillus* strains, such as *L. plantarum* [37,38]. Also, heterologous glucanase genes (from *Bacillus macerans*, *Bacillus subtilis*, *Clostridium thermocellum* and *Fibrobacter succino*) have been expressed in intestinal *Lactobacillus* strains, and the enzyme activity was from 0.5 nmol/mL to 1.66 μmol/mL [15,17,26,39,40]. The enzyme activity of heterologous phytase genes from *Aspergillus ficuum* expressed in *Lactobacillus* was up to 22.12 U/mL [20]. The enzyme activity data of the phytase gene from *B. subtilis* expressed in *Lactobacillus* were provided by Kerovuo and Tynkkynen (2001) [21].

In the current study, transformed *L. reuteri* supplementation of a barley-wheat-based diet only improved the feed conversion ratio from 21 to 42 days of age and for the whole feeding period but did not improve body weight gain or feed intake. A reduction of 0.1% of the available phosphorus content in broiler chicken rations is unlikely to significantly decrease the weight gain [41,42]. Yu *et al.* (2008) demonstrated that addition of *L. reuteri* Pg4 expressing heterologous β-glucanase to a barley-based diet only improved body weight gain from 22 to 37 days of age and from 0 to 37 days of age but did not improve feed intake or the feed conversion ratio [43]. Liu *et al.* (2007) found that supplementing a wheat-based diet with *L. reuteri* Pg4 expressing rumen fungal xylanase also only improved body weight gain from 0 to 21 days of age but not body weight gain from 21 to 42 days, feed intake or feed conversion ratio over the whole feeding period [26]. Jin *et al.* (1998) showed that supplementation with *L. acidophilus* I-26 strain or a mixture of 12 *Lactobacillus* cultures to broiler diets significantly improved body weight gain and feed conversion ratio in broilers from 0 to 6 weeks [44]. Askelson *et al.* (2013) reported that the body weight of chicks administered *Lactobacillus* *gasseri TDCC 65* expressing phytase was the same as the positive control group at days 14 and 21 [45]. However, Watkins and Miller (1983) showed that addition of *Lactobacillus* spp. cultures to broiler diets did not significantly improve growth performance [46]. It was, therefore, suggested that the use of different strains and dosages, different methods of preparation and the health status of chicks could produce dissimilar results [43].

The concentrations of ash, calcium and phosphorus in poultry tibiae are important parameters to measure minerals reserves, bone strength and bioavailability of calcium and phosphorus. In the present study, ash, calcium and phosphorus contents of tibiae in the negative control group were significantly lower than those in the positive control group, which suggested that low phosphorus levels in the diet significantly reduced the salinity of the tibia and the deposition of calcium and phosphorus in the tibia. In the current study, administration of *L. reuteri* pLEM4157 (cel), *L. reuteri* pLEM4158 (phy) and *L. reuteri* pLEM4159-cel/phy to a barley-wheat-based diet improved the ash, calcium and phosphorus contents of tibiae at day 21 and only the phosphorus level at day 42. Similarly, Perney *et al.* (1993) reported that the addition of dietary phytase to a maize-soybean meal diet with inadequate phosphorus did not improve body weight gain or feed intake, but it increased toe and tibia ash and plasma inorganic phosphorus contents in broiler chickens [47]. Leeson *et al.* (2000) also observed that phosphorus levels in tibia ash of chicks administered a phytase-supplemented diet were increased [48]. In contrast, Ahmad *et al.* (2000) showed that phytase supplementation did not cause any change in the content of calcium or phosphorus in tibia ash [49]. Sebastian *et al.* (1998) reported that the content of intestinal phytase was very low in young birds but increases with age, and dietary calcium content and the ca1cium:phytate ratio may be important factors that determine the extent of phytate hydrolysis [50]. Tibia ash and calcium and phosphorus levels are, therefore, likely to be influenced by many factors.

Different gastrointestinal tract regions of chickens play different roles in feed digestion, nutrient absorption and intestinal health, all of which depends on the ecological balance of the different microorganisms in the different gastrointestinal tract regions [51]. Augmentation of intestinal coliform numbers may result from an imbalance in the ratio of probiotics to potentially pathogenic bacteria in the gastrointestinal tract [52]. In the present study, the counts of *E. coli*, *Veillonella* spp. and *B. vulgatus* in the cecum of broilers administered transformed *L. reuteri* indicated a lower trend than the control groups. An opposite trend was observed for the counts of *Bifidobacterium* genus and* Lactobacillus* spp*.* at day 21. Similar results were observed by Jin *et al.* (1998), Mathlouthi *et al.* (2002), and Yu *et al.* (2007) [43,44,53]. It may be speculated that *Lactobacillus* spp. may depress the growth of intestinal coliform bacteria by releasing organic acids or inhibin and by competing with pathogenic bacteria for adhesion loci. Exogenous enzymes may further inhibit the growth of intestinal coliform bacteria via oligosaccharides, which are released after hydrolysis of non-starch polysaccharides and are fermented by specific bacteria to decrease pH values [44,54].

## 4. Materials and Methods

### 4.1. Bacterial Strains, Plasmids, and Growth Conditions

The strains and the plasmids used in the current study are listed in Supplementary Table S1. *E. coli* strains were selected on a Luria–Bertani (LB) plate and cultured in LB broth with shaking at 250 rpm and 37 °C. The *L. reuteri* strains were selected on a Man, Rogosa, and Sharpe (MRS) plate and cultured in MRS broth under anaerobic conditions at 37 °C. The following antibiotics concentrations were used for selection: 100 μg/mL of ampicillin (Sigma, San Diego, CA, USA) or 5 μg/mL of erythromycin (Sigma, San Diego, CA, USA).

### 4.2. DNA Isolation and Manipulation

All DNA manipulations were performed according to standard procedures described by Sambrook *et al.* (2011) [55]. *B. subtilis* WL001 (GenBank No. KJ528401) and *A.* *fumigatus* WL002 (GenBank No. KJ528402) genomic DNAs were isolated using the Bacteria DNA Kit (Omega Bio-Tek, Doraville, CA, USA) and the Biospin Fungus Genomic DNA Extraction Kit (BioFlux Bioer Technology, Tokyo, Japan), respectively, according to the manufacturers’ instructions. *E. coli* and *Lactobacillus* plasmid DNA were isolated with the Plasmid DNA Mini Kit II (Omega Bio-Tek, Doraville, CA, USA) according to the protocol provided by the manufacturer (Omega Bio-Tek, Doraville, CA, USA) with some modifications (cell sediment was washed by sterile water 3 times, then 250 μL Solution I and 20 μL 25 mg/mL lysozyme was added and incubated at 37 °C for 10–30 min). Restriction enzymes and T4 DNA ligase (TaKaRa, Dalian, China; Promega, Madison, WI, USA) were used according to the manufacturer’s instructions.

### 4.3. Construction of Coexpression Vector

The fusion fragment containing lactate dehydrogenase promoter *P_IdhL_*, *Usp45* gene signal peptide *SP_usp45_* and enhancer *LEISS * was codon-optimized and synthesized by Genscript Bio-Technology (Nanjing, China) based on their amino acid sequences (GenBank No. M76708.1; M60178.1) and codon bias of *L. lactis* [24]. *BamH*I and *Spe*I restriction sites were added to the 5' and 3' ends of the fragment, respectively. Then the fusion fragment was digested with *BamH*I and *Spe*I and ligated with *BamH*I–*Spe*I-digested pLEM415 [56] to generate pLEM4155. The DNA sequence harboring the ribosome binding site *rbs*, *usp45* gene signal peptide *SP_usp45_* and enhancer *LEISS* was amplified by PCR from pLEM4155 using the primer pair G1/G2 (Supplementary Table S2) with the introduction of a 5' *Xba*I site and a 3' *Not*I site. The amplified fragment was digested with *Xba*I and *Not*I and ligated with *Xba*I–*Not*I cut pLEM4155 to generate the coexpression vector pLEM4156.

### 4.4. Construction of Endoglucanase and Phytase Coexpression Plasmid

The endoglucanase gene *celW* (GenBank No. KJ528404) and phytase gene mature peptide *phyWM* (GenBank No. KJ528403) were amplified by PCR from the chromosomal DNA of *B. subtilis* WL001 and *A.* *fumigatus* WL002, respectively. The primer pairs C1/C2 and P1/P2 (Table 2) were used to amplify *celW* and *phyWM* genes, respectively, and designed to insert a *Spe*I site at the 5' end and a *Xba*I site at the 3' end of the PCR products. These were then digested with *Spe*I and *Xba*I and ligated with *Spe*I–*Xba*I-digested pLEM4155 to generate pLEM4157 (cel) and pLEM4158 (phy), respectively. The primer pair P3/P4 (Supplementary Table S2) was used to amplify the phyWM gene and to introduce a *Not*I site at the 5' end and a *Sac*II site at the 3' end of the PCR product. *Not*I–*Sac*II-digested *phyWM* PCR product and *Spe*I–*Xba*I-digested *celW* PCR product were ligated with pLEM4156 to generate pLEM4159-cel/phy.

### 4.5. Transformation of Plasmid DNA

Competent *E. coli* cells were prepared and electroporated by the standard methods of Walker and Klaenhammer (1994) [57]. The transformants were selected on LB agar plates containing ampicillin (100 μg/mL, Sigma, San Diego, CA, USA). The coexpression vector plasmids containing endoglucanase and phytase gene were electroporated into *L. reuteri* XC1 (GenBank No. KJ528405) using the method described by Serror *et al.* (2002) [56]. After electroporation, the *L. reuteri* XC1 transformants were incubated in MRS broth containing 10 mM MgCl_2_ at 37 °C for 3 h, then spread on MRS agar plates containing erythromycin (5 μg/mL, Sigma, San Diego, CA, USA) and incubated at 37 °C for 48 h. The transformants were identified by PCR amplification using the two primer pairs (C1/C2 and P1/P2), and screened on MRS agar plates containing 0.1% CMC (Sigma, San Diego, CA, USA) by the Congo red dye method [58] or on MRS agar plates containing 0.5% sodium phytate (Sigma, San Diego, CA, USA) by 2% (*w*/*v*) cobalt chloride solution staining and aqueous cobalt chloride solution and an ammonium molybdovanadate solution for counterstaining. Colonies forming yellow halos and hyaline halos were selected for further analysis.

### 4.6. Determination of Endoglucanase and Phytase Activity

The *L. reuteri* XC1 transformants identified above were inoculated in Man, Rogosa, and Sharpe (MRS) broth containing erythromycin (5 μg/mL). After incubation at 37 °C for 48 h, the fermented broths of transgenic *L. reuteri* XC1 were centrifuged to separate and collect culture supernatant and cell sediment. Culture supernatant was used to determine extracellular enzyme activity. The cell pellets were resuspended in 50 mM sodium citrate buffer (pH 5.0) and sonicated for 10 min to collect cell lysates for assaying intracellular enzyme activity [17]. Phytase and endoglucanase in the supernatant were determined with a previously described protocol [1,2]. One phytase unit was defined as the activity that released 1 μmol of inorganic phosphorous from sodium phytate per minute under the assay conditions. One unit of the fibrolytic enzyme activity was defined as the activity that released 1 μmol of reducing sugar from carboxymethyl cellulose (CMC) per minute under the assay conditions.

### 4.7. Sodium Dodecyl Sulfate Polyacrylamide Gel Electrophoresis (SDS-PAGE) Analysis

Culture supernatant collected above was concentrated by trichloroacetic acid (TCA) protein precipitation protocol [59] and then boiled with 4× sample buffer and analyzed by SDS-PAGE gel electrophoresis.

### 4.8. Broiler Chickens, Diet and Growth Trail

A total of 480 1-day-old Arbor Acres (AA) male broilers with similar body weight were assigned to six treatment groups and allocated to 24 cages, each cage containing 20 chicks. The six groups were offered six dietary treatments of barley-wheat-based diets with four replicates for each treatment. Preparation of recombinant *L. reuteri* was performed according to the method described by Mappley *et al.* (2013) [60]. Control groups were fed a phosphorus adequate diet (as positive control) or phosphorus deficient diet (as negative control) and administered distilled water from day 1. Four experimental treatment groups were fed a phosphorus deficient diet and administered distilled water containing recombinant *L. reuteri* pLEM4156, *L. reuteri* pLEM4157 (cel), *L. reuteri* pLEM4158 (phy), and *L. reuteri* pLEM4159-cel/phy (2.5 × 10^8^ cfu/mL) from day 1, respectively. The mixture was included in the water container and water was freely accessible to all birds and changed frequently to keep fresh. Feeds were supplied for consumption *ad libitum*. The composition of the experimental diet is presented in Supplementary Table S3. Chicks were raised under controlled management similar to commercial practice during the experimental period (0–42 days). Body weight and feed intake were recorded at ages days 21 and 42, and the feed conversion ratio (feed intake:weight gain) was calculated. The experimental protocol was approved by the Institutional Experimental Animal Care and use Committee of Northwest A&F University and performed in according with animal welfare and ethics.

### 4.9. Determination of Tibia Parameters

At age days 21 and 42, two birds were randomly selected from each cage and killed by cutting the carotid arteries, and then the left tibia was removed and frozen. Tibia were cleaned for 48 h with ethyl alcohol and for another 48 h with ethyl ether, and dried for 24 h at 110 °C. After weighing, tibiae were turned to ash in a muffle furnace overnight at 600 °C and ash, calcium and phosphorus content were subsequently measured. The tibia length was measured using a vernier caliper.

### 4.10. Analysis of Microbial Distribution in the Cecum by Real-Time Quantitative PCR

Genomic DNA from cecum content was isolated using a Stool DNA Kit (Omega, Doraville, CA, USA) according to the manufacturer’s instructions, and DNA concentration and purity were determined using a NanoDrop 1000 Spectrophotometer (Thermo Fisher Scientific, San Jose, CA, USA). Samples were stored at −20 °C.

The different species-specific primers (listed in Supplementary Table S4) for quantitating microbes were adapted from published papers and were checked for specificity by nucleotide-nucleotide BLAST searches of the NCBI database. The specific regions of target bacteria were amplified by conventional PCR using the different species-specific primers and genomic DNA from chicken gastrointestinal tract contents. Amplicons were purified using agarose gel electrophoresis and a Gel Extraction Kit (Omega, Doraville, CA, USA) and then inserted into the pGEM-T easy vector (Promega, Madison, WI, USA) and sequenced by Genscript bio-Technology, Nanjing, China to confirm the recombinant plasmids. The concentration and number of copies of the correct recombinant plasmids were determined. The standard plasmids were obtained from serial 10-fold dilutions (from 10^3^ to 10^9^) of recombinant plasmids, and then real-time quantitative PCR was performed to construct a standard curve (Supplementary Table S5) with the threshold cycle (*C*_t_) corresponding to the number of copies.

Real-time quantitative PCR was performed with the iCycler iQ real-time detection system (Bio-Rad, Hercules, CA, USA) using 96-well optical-grade PCR plates. The PCR was performed in triplicate in a total volume of 25 μL using SYBRII Green PCR Master mix (Takara, Dalian, China). Each reaction included 150 ng of template DNA, 12.5 μL of SYBRII Green PCR Master mix and 2 mM of each primer. The reaction conditions were set as follows: 50 °C for 2 min, 95 °C for 10 min, 40 cycles of 95 °C for 15 s and 60 °C for 1 min. Melting curve: 81 cycles of 55 °C for 30 s, temperature change 0.5 °C, end temperature 95 °C. Data analysis was carried out with iCycler Optical System Interface software (version 2.3; Bio-Rad, Hercules, CA, USA).

### 4.11. Statistical Analysis

Data were subjected to one-way analysis of variance analysis using SPSS13.0 software (SPSS Inc., Chicago, IL, USA) for variance analysis and significance testing. Duncan’s method was used to test differences among different treatments.

## 5. Conclusions

In conclusion, the *L. reuteri* pLEM4158-cel/phy-transformed strain successfully displayed the ability to hydrolyze carboxymethyl cellulose (CMC) and calcium phytate. Transformed *L. reuteri* supplementation of a barley-wheat-based diet improved the feed conversion ratio from 21 to 42 days of age and over the whole feeding period but did not improve body weight gain or feed intake of chicks. It also improved the ash, calcium and phosphorus contents of tibiae at day 21 but only phosphorus contents at day 42. Furthermore, counts of *E. coli*, *Veillonella* spp. and *B. vulgatus* in the cecum were decreased, while counts of *Bifidobacterium* genus and *Lactobacillus* spp. at day 21 were increased *.*

## Supplementary Files

Supplementary File 1
