# Supplementary material for: Coexpression and Secretion of Endoglucanase and Phytase Genes in Lactobacillus reuteri
Source: Int J Mol Sci. 2014 Jul 21;15(7):12842–60. doi: 10.3390/ijms150712842 (PMC4139877; doi:10.3390/ijms150712842)
Supplement: Supplementary File 1 [file ijms-15-12842-s001.pdf]

Supplementary Information

Table S1. Bacterial strains and plasmids used in this study.

Strain/Plasmid	Characteristics	Source/Reference
**Strain**		
E. coli DH5α	F-φ80d lacZΔM15Δ(lacZYA-argF) U169 endA1 recA1 hsdR17 (r_k^-, m_k^+) supE44λ-thi-1 gyrA96 relA1 phoA	TransGen, Beijing, PRC
B. subtilis WL001	wild type, isolated from soil	isolated by our research group
A. fumigatus WL002	wild type, isolated from soil	isolated by our research group
L. reuteri XC1	Host strain isolated from chick gut	isolated by our research group
L. reuteri pLEM 4156	Carrying plasmid pLEM4156	this work
L. reuteri pLEM 4157(cel)	Carrying plasmid pLEM4157 (cel)	this work
L. reuteri pLEM 4158 (phy)	Carrying plasmid pLEM4158 (phy)	his work
L. reuteri pLEM 4159-cel/phy	Carrying plasmid pLEM4159-cel/phy	this work
**Plasmid**		
pLEM415	Amp ^{r}, Em ^{r}; *E. coli–L. reuteri* shuttle vector; 6.3 kb	[1]
pLEM4155	the fusion fragment of IdhL promoter, *Usp45* signal peptide and enhancer cloned into pLEM415; 6.6 kb	this work
pLEM4156	the PCR fragment containing the ribosome binding site (*rbs*) fusing to Usp45 gene signal peptide (SPusp45) and enhancer (LEISS) cloned into pLEM4156; 6.7 kb	this work
pLEM4157 (cel)	*B. subtilis* WL001 endoglucanase gene *cel15* cloned into pLEM4155; 8.1 kb	this work
pLEM4158 (phy)	*A. fumigatus* WL002 phytase gene mature peptide *phyWM* cloned into pLEM4155; 7.9 kb	this work
pLEM4159-cel/phy	*phyWM* and *celW* cloned into pLEM4156; 9.6 kb	this work

Table S2. Primers used in the work.

Name	Sequence(5'–3') [a,b]
G1	TGCTCTAGA**GAAAGGA**TGATATCACCATGCAATCAAGTTTAAAGAAAT (*Xba*I)
G2	TTTGCGGCCGCAGCATCACATGTTGATGAGATTTC (*Not*I)
C1	CGGACTAGTATGAAACGGTCAATCTCGATTTT (*Spe*I)
C2	TGCTCTAGACTAATTTGGTTCTGTTCCCCAAATCA (*Xba*I)
P1	CGGACTAGTTCCAAGTCCTGCGATACGGTAGACCTC (*Spe*I)
P2	TGCTCTAGATCAACTAAAGCACTCTCCCCAGTTGCC (*Xba*I)
P3	TTTGCGGCCGCGTCCAAGTCCTGCGATACGGTAGACCTC (*Not*I)
P4	TCCCCGCGGTCAACTAAAGCACTCTCCCCAGTTGCC (*Sac*II)

[a] The nucleotide sequence underlined was restriction site; and [b] The nucleotide sequence in bold was ribosome binding site *rbs*.

Table S3. Composition of the experimental diet.

Parameter	Days 1–21		Days 21–42	
	Positive Control	Negative Control	Positive Control	Negative Control
**Composition (%)**				
Barley	39.06	38.22	41.00	40.00
Wheat	30.00	30.00	31.00	28.78
Soybean meal	18.00	20.00	12.54	17.05
Fish meal	6.54	5.20	6.87	4.00
Soybean oil	3.39	3.54	5.29	5.76
Limestone	1.09	1.46	1.60	1.85
Dicalcium phosphate	0.55	0.17	0.20	0.10
Salt	0.16	0.19	0.28	0.21
Choline-Cl, 50%	0.10	0.10	0.10	0.10
Methionine	0.10	–	0.03	0.05
Lysine	–	0.01	0.10	0.10
1% Premix [1]	1.00	1.00	1.00	1.00
**Nutrient level**				
Metabolizable Energy (kcal/kg)	2900	2900	3041	3041
Crude Protein (%)	21.00	21.00	19.00	19.00
Calcium (%)	0.90	0.90	1.00	0.96
Total Phosphorus (%)	0.65	0.56	0.58	0.50
Available Phosphorus (%)	0.45	0.35	0.40	0.30

[1] The premix provided per kilogram of diets: iron, 100 mg; zinc, 100 mg; copper, 8 mg; manganese, 120 mg; iodine, 0.7 mg; and selenium, 0.3 mg; vitamin A, 8000 IU; vitamin D3, 1000 IU; vitamin E, 20 IU; menadione, 0.5 mg; thiamine, 2.0 mg; flavin, 8.0 mg; niacin, 35 mg; pyridoxine, 3.5 mg; vitamin B12, 0.01 mg; pantothenic acid, 10.0 mg; folic acid, 0.55 mg; biotin, 0.18 mg; antioxidant, 0.4 g in the phase of 1–21 days, and iron, 60 mg; zinc, 80 mg; copper, 8 mg; manganese, 60 mg; iodine, 0.6 mg; and selenium, 0.3 mg; vitamin A, 6000 IU; vitamin D3, 500 IU; vitamin E, 30 IU; menadione, 0.5 mg; thiamine, 2.0 mg; flavin, 5.0 mg; niacin, 30 mg; pyridoxine, 3.0 mg; vitamin B12, 0.01 mg; pantothenic acid, 10.0 mg; folic acid, 0.55 mg; biotin, 0.15 mg ; antioxidant, 0.5 g in the phases of 22–42 days.

Table S4. Real-time PCR primers.

Species	Primers	Sequence (5'–3')	Amplicon Length (bp)	Reference
Escherichia coli	F col	GTTAATACCTTTGCTCATTGA	340	[2]
	R col	ACCAGGGTATCTAATCCTGTT		
Bifidobacterium genus	F bif	GGGTGGTAATGCCGGATG	442	[3]
	R bif	TAAGCCATGGACTTTCACACC		
Bacteroides vulgatus	R bac	AAGGGAGCGTAGATGGATGTTTA	193	[4]
	F bac	CGAGCCTCAATGTCAGTTGC		
Veillonella spp.	F vei	AYCAACCTGCCCTTCAGA	343	[3]
	R vei	CGTCCCGATTAACAGAGCTT		
Clostridium IV	F clo	TTACTGGGTGTAAAGGG	584	[3]
	R clo	TAGAGTGCTCTTGCGTA		
Lactobacillus spp.	F lac	AGCAGTAGGGAATCTTCCA	341	[5]
	R lac	CACCGCTACACATGGAG		
Enterococcus faecalis	F ent	AACCTACCCATCAGAGGG	357	[6]
	R ent	GACGTTCAGTTACTAACG		

Table S5. The standard curve, PCR efficiency (E%) and linear correlation coefficient (R^2).

Species	***Standard Curve***	R^2	***E%***
Escherichia coli	$Y = -3.150X + 33.605$	0.996	107.7
Bifidobacterium genus	$Y = -3.251X + 32.545$	0.996	103.0
Bacteroides vulgatus	$Y = -3.109X + 33.103$	0.996	109.7
Veillonella spp.	$Y = -3.355X + 34.021$	0.997	98.6
Clostridium XIV	$Y = -3.632X + 36.06$	1.000	88.5
Lactobacillus spp.	$Y = -2.784X + 31.451$	0.985	110.7
Enterococcus faecalis	$Y = -3.142X + 34.182$	0.995	108.1

References

1. Ref. [1] is the mentioned in the main text and the order is ref. [56].
2. Malinen, E.; Kassinen, A.; Rinttilä, T.; Palva, A. Comparison of real-time PCR with SYBR Green I or 5'-nuclease assays and dot-blot hybridization with rDNA-targeted oligonucleotide probes in quantification of selected faecal bacteria. *Microbiology* **2003**, *149*, 269–277.
3. Wise, M.G.; Siragusa, G.R. Quantitative analysis of the intestinal bacterial community in one-to three-week-old commercially reared broiler chickens fed conventional or antibiotic-free vegetable-based diets. *J. Appl. Microbiol.* **2007**, *102*, 1138–1149.
4. Huijsdens, X.W.; Linskens, R.K.; Mak, M.; Meuwissen, S.G.; Vandenbroucke-Grauls, C.M.; Savelkoul, P.H. Quantification of bacteria adherent to gastrointestinal mucosa by real-time PCR. *J. Clin. Microbiol.* **2002**, *40*, 4423–4427.
5. Štšepetova, J.; Sepp, E.; Kolk, H.; Loivukene, K.; Songisepp, E.; Mikelsaar, M. Diversity and metabolic impact of intestinal *Lactobacillus* species in healthy adults and the elderly. *Br. J. Nutr.* **2011**, *105*, 1235–1244.
6. Bartosch, S.; Fite, A.; Macfarlane, G.T.; McMurdo, M.E. Characterization of bacterial communities in feces from healthy elderly volunteers and hospitalized elderly patients by using real-time PCR and effects of antibiotic treatment on the fecal microbiota. *Appl. Environ. Microbiol.* **2004**, *70*, 3575–3581.
